# Construction and Characterization of Three Wheat Bacterial Artificial Chromosome Libraries

**DOI:** 10.3390/ijms151221896

**Published:** 2014-11-28

**Authors:** Wenjin Cao, Bisheng Fu, Kun Wu, Na Li, Yan Zhou, Zhongxia Gao, Musen Lin, Guoqiang Li, Xinyi Wu, Zhengqiang Ma, Haiyan Jia

**Affiliations:** College of Agricultural Sciences, Nanjing Agricultural University, Nanjing 210095, China; E-Mails: caowenjin83@gmail.com (W.C.); bishengfu@126.com (B.F.); kunwu82@126.com (K.W.); nali310312@126.com (N.L.); yanzhou89@126.com (Y.Z.); zhongxiagao88@126.com (Z.G.); musenlin88@126.com (M.L.); Guoqiangli82@126.com (G.L.); xinyiwu88@126.com (X.W.); zqm2@njau.edu.cn (Z.M.)

**Keywords:** bacterial artificial chromosome, wheat, descending pool

## Abstract

We have constructed three bacterial artificial chromosome (BAC) libraries of wheat cultivar *Triticum aestivum* Wangshuibai, germplasms *T. monococcum* TA2026 and TA2033. A total of 1,233,792,170,880 and 263,040 clones were picked and arrayed in 384-well plates. On the basis of genome sizes of 16.8 Gb for hexaploid wheat and 5.6 Gb for diploid wheat, the three libraries represented 9.05-, 2.60-, and 3.71-fold coverage of the haploid genomes, respectively. An improved descending pooling system for BAC libraries screening was established. This improved strategy can save 80% of the time and 68% of polymerase chain reaction (PCR) with the same successful rate as the universal 6D pooling strategy.

## 1. Introduction

Wheat is one of the most important food crops in the world [1]. The lack of a complete genome sequence limits the research available of the wheat genome. Under these circumstances, a large-insert genome library is critical for physical mapping, map-based gene cloning, whole-genome sequencing, genome structure analysis, and evolutionary studies within grass species [2]. Bacterial artificial chromosome (BAC)-based sequencing presents advantages over the use of whole-genome shotgun methods for wheat physical map construction and whole genome sequencing [3], owing to the high content of repetitive elements of wheat genome [4,5].

BAC libraries of small-genome grass species such as rice have been used for comparative mapping and gene cloning in wheat [6,7,8,9]. However, this strategy could be adventurous because of genome rearrangement and evolution. Hence, BAC libraries have been constructed from genomic DNA of *Triticeae* species including *T.* *urartu*, *Ae.* *speltoides* and *Ae*. *tauschii* [10], *T*. *tauschii* [11], *T. monococcum* [12], durum wheat [13], and common wheat [14,15] *et al*. However, the evolutionary divergence within species and genomes [16,17] hinders the application of these BAC libraries, especially for the resistant genes, which evolved rapidly to adapt to different environments. For example, to clone genes resistant to powder mildew and Fusarium head blight (FHB), which are the main diseases during wheat growth, specific resistance germplasm libraries were strongly needed.

Of the methods of BAC library screening, Southern blotting is favored for species with small genome sizes, such as Arabidopsis [18] and rice [19]. Common wheat has a large genome size (≈17 Gb) [20], and it is time-consuming and impractical to screen full genome libraries by filter hybridization. High-density membranes have also been prepared for di-, tetra- and hexaploid wheat whole-genome and chromosome-specific libraries [12,13,21,22]. The large number of membranes also limits the practicality of BAC libraries screening in common wheat. For example, 18,432 clones should be spotted on one 22.2 × 22.2 cm^2^ membrane. Thus, 69 membranes were prepared for the screening of the Norstar library containing 1,266,432 clones [21]. Polymerase chain reaction (PCR) based 6D [23,24,25], 5D [26,27], and other multi-dimensional [15,28] BAC pooling strategies have been used in BAC libraries screening. All of these methods were complex and time-consuming, therefore, an effective and simple method was needed.

In this study, we describe the construction of three BAC libraries of resistant wheat germplasms. Based on that, an improved descending pooling system for BAC library screening is developed and applied.

## 2. Results and Discussion

### 2.1. Construction of Three Bacterial Artificial Chromosome Libraries

To optimize the conditions for ligation, we prolonged the ligation time and compared the ligation time with that for a 1-day reaction, as described in the Section Materials and Methods. The transformation efficiencies of the 3-day ligation reactions with 1.5–5.1 × 10^5^/μg DNA were 6.8–9.2 times higher than the efficiency of 1-day ligation (Table 1) and 1.5 to 5 times higher than the 1 × 10^5^/μg DNA of the wheat BAC library of DV92 constructed by Lijavetzky *et al.* [12]. The percentage of empty clones decreased when ligation time was prolonged (Table 1). These results showed that the prolonged ligation reaction significantly increased transformation efficiency and that fewer empty clones were observed (Table 1), which was in contrast to the result of Osoegawa *et al*. [29], although the mechanism was unknown. It was reported that the transformation efficiency decreased to 18-fold in wheat, whereas the insert size increased [21]. The prolonged ligation reduced this problem.

**Table 1:** Comparison between 1- and 3-day ligation reactions.

Repeat	Transformation Efficiency (×10^4^/μg DNA)		Average Insert Size (Kb)		Empty Clones (%)	
	1-Day	3-Day	1-Day	3-Day	1-Day	3-Day
Repeat 1	2.25	15.4	88	131	7.07	2.70
Repeat 2	6.45	45.1	114	138	3.21	0.14
Repeat 3	5.57	51.5	108	149	3.34	0.04

A BAC library of common wheat Wangshuibai and two diploid wheats, TA2026 and TA2033, was constructed according to the procedures described above. Cloning bias owing to non-uniform distribution of restriction sites [30] may cause the failure of cloning target fragments to be described as a vector. To compensate for this heterogeneous distribution of the restriction enzyme sites on the genome [31], *Bam*HI and *Hin*dIII were used to digest High-molecular-weight (HMW) DNA of Wangshuibai and TA2033, whereas *Bam*HI was used for the construction of the TA2026 library. Thus, libraries of Wangshuibai and TA2033 were composed of two parts, *Bam*HI and *Hin*dIII.

As a result, the numbers of clones picked for Wangshuibai, TA2026, and TA2033 were 1,233,792 (596,736 of *Bam*HI and 637,056 of *Hin*dIII), 170,880, and 263,040 (183,936 of *Bam*HI and 79,104 of *Hin*dIII), respectively. All of these clones were stored in 3213 (1554 of *Bam*HI and 1659 of *Hin*dIII), 445 and 685 (479 of *Bam*HI and 206 of *Hin*dIII) 384-well plates. There were a total of 1,667,712 clones in the three libraries. All BAC clones were employed in the construction of descending pools as described in the Materials and Methods Section. In total, 530 (260 of *Bam*HI and 270 of *Hin*dIII), 74, and 114 (80 of *Bam*HI and 34 of *Hin*dIII) tertiary pools were constructed for the libraries of Wangshuibai, TA2026 and TA2033, respectively.

### 2.2. BAC Library Characterization

#### 2.2.1. Average Insert Size

**Figure 1:**
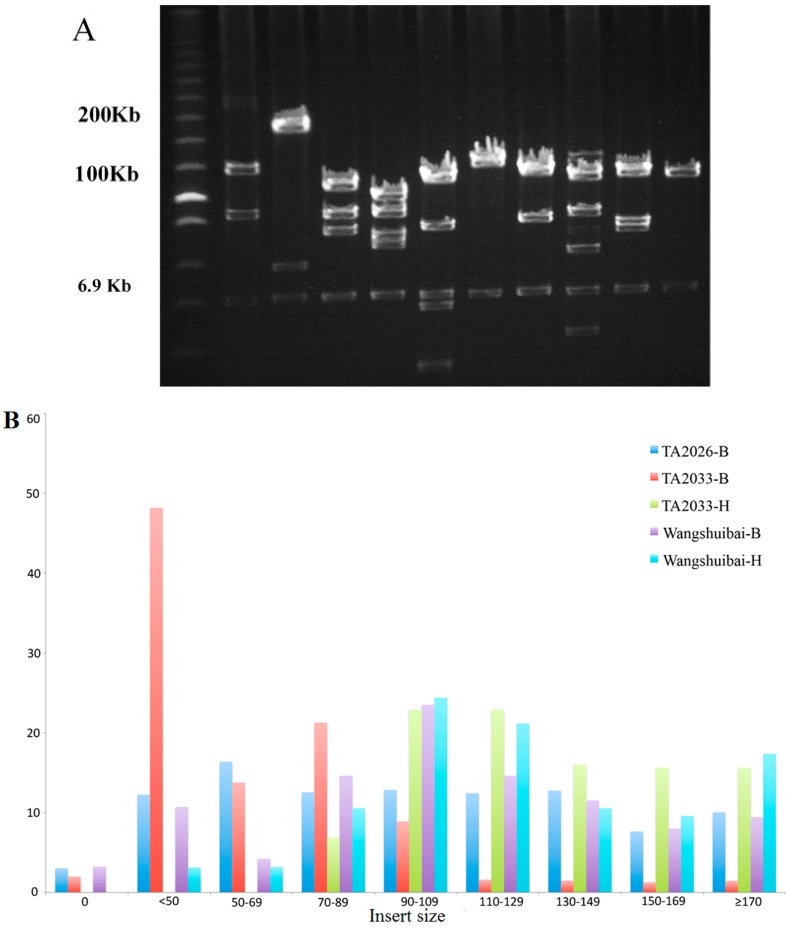
Insert size distribution of bacterial artificial chromosome (BAC) libraries. (**A**) Pulsed-field gel electrophoresis (PFGE) of 10 randomly selected clones from TA2033 (*Bam*HI) library. BACs isolated from *E. coli* were digested with *Not*I and separated on 1% agarose gel with a ramp pulse time of 1–40 s at 6 V/cm at 12.5 °C in 0.25× TRIS-Borat-EDTA (TBE) buffer for 16 h. The marker is Low Range PFG marker (#N0350S, New England Biolabs, Ipswich, MA, USA). Bands of vector PindigoBAC-5 are indicated as 6.9 kb by the arrowhead. Sizes of individual BAC clones are calculated relative to the DNA size markers; (**B**) Insert size distribution (percentage, %) of all randomly selected clones of five libraries. -B and -H indicate digestion with *Bam*HI and *Hin*dIII, respectively. To estimate the insert size distribution, 155 (111 of -*B* and 44 of -*H*), 298 and 185 (152 of -*B* and 33 of -*H*) clones from Wangshuibai, TA2026, and TA2033 libraries were analyzed.

To determine the average insert size of the three libraries, 155 (111 of *Bam*HI and 44 of *Hin*dIII), 298, and 185 (152 of *Bam*HI and 33 of *Hin*dIII) clones were selected from the Wangshuibai, TA2026, and TA2033 libraries, respectively. The insert DNA fragments were released from the vector with enzyme *Not*I followed by pulsed-field gel electrophoresis (PFGE) to determine the average insert size of each set of clones (Figure 1A).

Empty clones were found in the Wangshuibai (*Bam*HI), TA2026, and TA2033 (*Bam*HI) libraries. The percentages were 3.2, 3.03, and 1.53, respectively. No empty clones were found in the libraries Wangshuibai (*Hin*dIII) and TA2033 (*Hin*dIII). The empty clones were included in the calculation of the average insert size of each set. As each of the three libraries originated from several sets of digested products, we multiplied the average insert size of each set by the proportion of the clones of the full library that it contained and then summed these products to calculate the final average insert size of the full library.

The average insert sizes of Wangshuibai (both *Bam*HI and *Hin*dIII) and TA2033 (*Hin*dIII) libraries were large, 124.49, 123.21, and 127.54 kb, respectively (Table 2, Figure 1B). TA2026 showed a moderate average insert size by 88.64 kb (Table 2, Figure 1B). Library TA2033-*Bam*HI had the smallest average insert size with 59.57 kb, because it had the largest proportion of clones with insert sizes less than 50 kb (Table 2, Figure 1B).

**Table 2:** Characteristics of the three BAC libraries.

BAC Library	Enzyme	Organelle DNA (%)	Empty Clones (%)	NO. of Clones	Average Insert size ^a^/min-max (Kb)	Genome Coverage (×) ^b^
TA2026	*Bam*HI	0.78	3.03	170,880	88.64/0-310	2.60
TA2033-*B*	*Bam*HI	0.78	1.53	183,936	59.57/15-285	1.91
TA2033-*H*	*Hin*dIII	0	0	79,104	127.54/80-224	1.80
Total	*-*	0.55	1.07	263,040	80.01	3.71
Wangshuibai-*B*	*Bam*HI	0	3.2	596,736	124.49/30-320	4.42
Wangshuibai-*H*	*Hin*dIII	0.78	0	637,056	123.21/20-265	4.63
Total	*-*	0.40	1.54	1,233,792	122.83	9.05

^a^ The empty clones were included in the calculation of average insert size; ^b^ Chloroplasts and mitochondria DNA and empty clones were excluded.

The average insert sizes of TA2026 and TA2033 (*Bam*HI) were 88.64 and 59.57 kb, respectively. Insertion with sizes as low as 4 kb appeared in the TA2026 library. Approximately a third of the ligation reactions of the TA2026 library and the entire TA2033 (*Bam*HI) library were constructed using DNA by one size selection. Clones with sizes as small as 5 kb were also found ligated to the pIndigoBAC-5 vector in another wheat BAC library when one size DNA selection was used to establish half of the ligation reactions [32]. This procedure of the single size selection may lead to contamination with small DNA fragments [11,30,32], and the double size selection removed most of the small fragments, resulting in clones larger than 120 kb in the Wangshuibai-*Bam*HI, -*Hin*dIII, and TA2033-*Hin*dIII libraries.

Subtracting the empty clones, the library Wangshuibai (both *Bam*HI and *Hin*dIII) had an average insert size of 129 kb and TA2026 and TA2033 (both *Bam*HI and *Hin*dIII) had smaller insert sizes of approximately 80 kb (Table 2).

#### 2.2.2. Contamination with Organelle DNA

Hybridization of seven probes to double-spotted clones on nylon membranes were used to determine the level of organelle DNA contamination. Eight plates (1, 1, 3, 2, and 1) were randomly chosen for Wangshuibai (*Bam*HI and *Hin*dIII), TA2026, and TA2033 (*Bam*HI and *Hin*dIII). There were clearly signals on the films from 0, 3, 9, 6, and 0 positive clones. The level of organelle DNA of all three libraries was less than 0.8% (Table 2). The low content of organelle contamination was similar to that of other wheat libraries [12,13,33], whose nuclei were also isolated according to Zhang *et al.* [34]. No signal was detected in the Wangshuibai (*Bam*HI) and TA2033 (*Hin*dIII) libraries, although this does not mean that there was no organelle DNA contamination in these two libraries. The absence of signal may be due to the low level of less than 0.26% (one in 384 clones). Intact nuclei were isolated and separated from organelle DNA by sucrose density gradient centrifugation and decreased the contamination by organelle DNA [34].

#### 2.2.3. Genome Coverage

Subtracting the BAC clones with organelle DNA insertion and the average insertion size of the three BAC libraries, the coverages of the Wangshuibai, TA2026, and TA2033 libraries were estimated to be 9.05×, 2.60×, and 3.71×, respectively (Table 2), taking the genome sizes to be 5.6 Gb/C for diploid wheat and 16.8 Gb/C for Wangshuibai. The Wangshuibai (*Bam*HI and *Hin*dIII together) library had greater larger genome coverage (9×) than reported hexaploid wheat libraries of the Chinese Spring library (9.3×) constructed by Allouis *et al*. [14].

The actual genome coverage is a key characteristic of the BAC library. To estimate the genome coverage, 303 (5.3× genome coverage), 74, and 114 descending tertiary pools of Wangshuibai, TA2026, and TA2033 were screened with the Simple Sequence Repeat (SSr) markers listed in Table 3 (GWM135 to BARC108 for TA2026 and TA2033, 18 markers for Wangshuibai). The 12 secondary pools were amplified to confirm the results when the PCR products of a corresponding tertiary pool did not show strong bands. Finally, 0–18, 0–2, and 0–4 positive tertiary pools of Wangshuibai, TA2026, and TA2033, were identified as positive pools. This resulted in identification by 94.44%, 57.14%, and 71.43% markers of at least one positive clone of Wangshuibai, TA2026, and TA2033, respectively. According to the average number of positive pools for all markers, the coverages estimated using PCR primers were 6×, 0.71×, and 1.57× of Wangshuibai (5.3× chosen), TA2026, and TA2033, respectively.

The genome coverage estimated by PCR for the libraries of TA2026 and TA2033 was much lower than that estimated by insert sizes and clone numbers. We may speculate on the reasons for this result as follows: Firstly, seven markers were used to screen the two libraries. We expected that this low sample size would increase the statistical error, resulting in a low frequency of recovery of positive clones. Secondly, there were restriction sites in the primer sequences or PCR products. Thus, the primers could not bind normally to templates or extend when the genomic DNA was digested during the construction of libraries, resulting in the failure of recovery of positive clones. For example, the forward primer of marker WMC153 harbors a *Hin*dIII recognition site, possibly causing the failure of the binding of the primer to positive BAC clones when the genome sequence was cut during the construction of the library. In fact, WMC153 recovered no positive clones of the TA2033 library, which was constructed with enzymes *Bam*HI and *Hin*dIII. Thirdly, because of the marked variation in the distribution of GC content in the genome [35], the enzymes chosen to construct the libraries may be distributed non-uniform over the genome [30]. This distribution may account for the difference between markers used to screen the libraries.

**Table 3:** Characterization of the genome coverage of the Wangshuibai library by PCR amplification.

	Marker	Positive Pool	Theoretical Probability	Actual Probability
	GWM135	8	-	-
	GWM95	6	-	-
	WMC153	5	-	-
	BARC78	2	-	-
	GWM293	3	-	-
	BARC3	4	-	-
	BARC108	3	-	-
	BARC61	0 ^a^	-	-
	BARC7	7	-	-
	GWM234	10	-	-
	GWM626	3	-	-
	BARC65	5	-	-
	BARC169	18	-	-
	BARC71	2	-	-
	BARC225	16	-	-
	BARC143	8	-	-
	BARC173	2		-
	BARC111	6	-	-
Average	-	6	99.52%	94.44%

^a^ Marker recovered no (zero) positive pools.

To improve the probability of recovery of positive clones, some effective measures should be taken. (1) More clones should be picked. Picking more clones results in larger genome coverage, in turn resulting in higher recovery probabilities; (2) Different enzymes should be chosen to avoid the corresponding potential non-uniform distribution of restriction sites; (3) Libraries should be screened with different primers. Primers used to screen libraries should be redesigned from the target loci to avoid the known restriction sites; (4) Libraries with two or more genetic backgrounds were preferred to facilitate the progress of genetic mapping, contig-assembly and map-based cloning [36].

The likelihood of finding any target DNA depends on the number of clones, the average insert size and the genome size (using the formula *p* = 1−e^N[ln(1 − I/GS)]^, N for clone numbers, I for insert size, and GS for genome size [37,38]. The coverage estimated by clone number and insert size resulted in a greater than 97% probability of recovering any sequence of interest in a single BAC clone of the Wangshuibai (99.98%), TA2033 (97.55%), and TA2026 (92.6%) libraries according to this formula. To determine the actual efficiency of recovery of positive clones, 34 locus-specific markers (data not show) located on 3B, 4B, 5A, and 2D and *Rht-1* and another 18 SSR markers (Table 3) were used to screen the entire Wangshuibai library (both *Bam*HI and *Hin*dIII). All of the primer pairs except BARC61 recovered at least one positive pool (Table 3) from the Wangshuibai library. This result was in accordance with the theoretical 98.98% probability.

### 2.3. Descending Pooling

In view of the very large genome of wheat and the clone number of our libraries (more than 1.66 million clones in three libraries; Table 2), a descending pooling strategy was developed to reduce the work of BAC library screening. To avoid potential competition between clones during the cell culture in one pool [39], we mixed plasmid DNA instead of bacterial cells together to make tertiary pools. The descending pooling strategy grouped twelve 384-well plates together, and the total amount of DNA in a tertiary pool ranged from 138 to 319 Mb (Table 2). The genome coverage of each tertiary pool (2304 clones) was approximately 0.016 for Wangshuibai and 0.04 for TA2026 and TA2033. This coverage ensured that one positive tertiary pool would correspond to a positive clone. The number of clones in the tertiary (super) pools should not be too small or too large. Fewer clones are uneconomical and excessive numbers of clones reduce the probability of positives, owing to competition between clones [39]. There are some BAC pools comprising nearly 4000 clones [32,40], and even up to 18,432 clones [21] in wheat. A total of 2304 clones per pool were shown to be optimal, with few false positive or false negative clones [25].

### 2.4. High Efficiency of Descending Pooling System

To obtain positive clones, different pooling strategies were raised in several libraries. Screen efficiency was the most important character of a pooling strategy. Comparison between the descending and 6D pooling strategies highlights the advantages and disadvantages. Two hundred and eighty-eight 384-well plates, which represent 0.6× of Wangshuibai genome, were chosen to do that. As described in the Section Material and Methods, 48 and 288 pools were made, and 576 and 288 plasmids were extracted according to descending and 6D systems, respectively. Markers WMC413 and BARC95 were used to do PCR amplification. Results are listed in Table 4. For instance, marker BARC95 hit six positive pools of the 6D pooling strategy, pools PP_18_, FP_7_, SP_9_, RP_24_, CP_26_, and DP_16_ showed positive bands. These results confirmed the address of the positive clone as (9,7,18). For the descending pooling strategy, the positive clone relative to marker BARC95 should theoretically be in TP_17_ as described in the Section Material and Methods. As shown in Table 4, only the TP_17_ of descending pools showed a positive band. The same results were observed when using marker WMC413. These perfect matches between the two different pooling strategies confirmed the high efficiency of the descending pooling strategy (Table 4).

**Table 4:** Comparison between 6D and descending pooling strategy.

Marker	6-D Pool						Descending Pool	
	PP	FP	SP	RP	CP	DP	TP
BARC95-2D	18 ^a^	7	9	24	26	16	17
WMC413-4B	47	45	5	43	3	1	47

^a^ The value of pools that show positive bands compare to positive control.

Another significant advantage of descending over 6D pooling strategies was timesaving. Eighteen days were used to prepare the 6D pool that consisted of 110,592 clones, while the descending pool was finished in 2 days, more than 80% of time was saved. A total of 289 and 92 PCR reactions were theoretically needed to fish a positive clone from the 110,592 clones in the 6D pooling strategy and the decending strategy, respectively, hence the PCR reactions were reduced by 68%.

Conclusively, the descending pooling system will save more time without any decrease of screening efficiency.

## 3. Experimental Section

### 3.1. Plant Material

Common wheat Wangshuibai (*Triticum* *aestivum* L.), an indigenous Chinese cultivar with a high level of scab resistance, and two more diploid wheat *Triticum* *monococcum* accessions, TA2026 and TA2033, were used for library construction. TA2026 and TA2033 were kindly provided by Dr. B.S. Gill (Wheat Germplasm Resource Center at Kansas State University, Manhattan, KS, USA). These two types of wheat harbor the powdery mildew resistance genes *pm2026* [41] and *mlm2033* [9].

### 3.2. HMW DNA Isolation and Partial Digest

Nuclei were isolated from 20 to 30 g etiolated young leaves, as described by Peterson *et al*. [42] and Zhang *et al.* [34]. High-molecular-weight (HMW) DNA was released from nuclei by proteinase K in lysis buffer (0.1 mg/mL proteinase K dissolved in 0.5 M EDTA, pH 9.1) at 50 °C for 48 h. The lysis buffer was changed after 24 h. Plugs (usually containing 5 to 6 μg undigested HMW DNA) were partially digested with *Bam*HI or *Hin*dIII. After digestion, size selection was first applied by PFGE separation for 16 h with a setting of 6 V/cm, a pulse time of 1 to 40 s, a temperature of 12.5 °C, and an angle of 120°, and then for 16 h with the settings of 6 V/cm, a pulse time of 3 to 5 s, a temperature of 12.5 °C, and an angle of 120° in 0.25× TBE buffer. Agarose gel containing DNA fragments in the size ranges of 100 to 200 kb and 200 to 400 kb was eluted into 350 to 450 μL 1× TRIS-Acetat-EDTA (TAE) buffer using a Bio-Rad model 422 Electro-Eluter (Bio-Rad, Hercules, CA, USA).

Then, to exclude the small fragments trapped in large fragments, two rounds of size selection were conducted. The concentration of recovered DNA from 5 to 6 μg HMW DNA following one size selection was 10 ng·μL^−1^, whereas two rounds of size selection reduced the concentration to 2–3 ng·μL^−1^. To absorb excess water from the DNA solution, 30% PEG 8000 (polyethylene glycol 8000) was employed. The minimal DNA concentration used for ligation was 3 ng·μL^−1^.

### 3.3. Optimizing Ligation Condition

To optimize the ligation conditions, we established two ligation reactions. One tube was incubated at 16 °C for 24 h followed by 4 °C for another 2 days (3-day ligation) and the other was incubated at 16 °C for 24 h only (1-day ligation). Then the ligation products were transformed into the complement cells, respectively. The insert size and clone number (transformation efficiency) were checked and compared between the two methods. The experiments were repeated three times with three different patches of DNA.

The eluted DNA was ligated to pIndigoBAC-5 vectors (Epicentre, Madison, WI, USA). The mole ratio of vector to insert DNA was 5–10:1. The ligation products were introduced into ElectroMAX™ DH10B™ cells (Invitrogen, Carlsbad, CA, USA) with a Gene Pulser Xcell™ Total System (Bio-Rad, Hercules, CA, USA) at 1.7 kV/cm, 200 Ω in a 0.1-cm cuvette (Bio-Rad, Hercules, CA, USA). Transformed cells were spread on LB Petri plates containing 12.5 μg·mL^−1^ chloramphenicol, 0.55 mol/L Isopropyl β-d-Thiogalactoside (IPTG) and 80 μg of X-Gal/mL [43]. White clones were picked with sterile toothpicks and manually arrayed in 384-well plates filled with 80 μL freezing LB media, containing 12.5 μg·mL^–1^ chloramphenicol. All 384-well plates were incubated at 37 °C overnight until the media became turbid. Two copies of all clones were made, one for storage and another for descending-pool preparation. Clones in 384-well plates were stored at −80 °C.

### 3.4. Checking Insert Size

Clones were cultured overnight in 3 mL liquid LB media containing 12.5 μg·mL^–1^ chloramphenicol. Plasmids were subsequently isolated and digested with the restriction enzyme *Not*I, which spanned the insert site. The digested plasmids were separated by PFGE. Band size was estimated on the basis of the Low Range PFG Marker (#N0350S, New England Biolabs, Ipswich, MA, USA). Clones without insert DNA were considered empty clones.

### 3.5. DNA Probes and Southern Blot

Seven organelle genome genes, including *ndhA*, *rbcL* (chloroplasts, [44]), *trnC*, *trnY*, *mttB*, *nad7*, and one mitochondria-specific sequence (mitochondria, [45]) that were evenly distributed in the chloroplast and mitochondria were used as probes for detection of organelle DNA.

Clones were double-spotted onto Hybond-N+ (Amersham, Pittsburgh, PA, USA) membranes with a 384-pin Microplate replicator (Boekel, Northbrook, IL, USA). The membranes were incubated with the colony side up on the LB agar media containing 12.5 μg·mL^–1^ chloramphenicol. After overnight incubation at 37 °C, the membranes were soaked sequentially in 10% sodium dodecyl sulfate (SDS) for 3 min, then in denaturation solution (0.5 M NaOH, 1.5 M NaCl) for 5 min, and then in neutralizing solution (1.5 M NaCl, 0.5 M Tris-HCl; pH 7.4) for 5 min twice. The membranes were then soaked in 2× SSPE buffer (300 mM NaCl, 20 mM NaH_2_PO_4_, 2 mM EDTA; pH 7.4) for 5 min. After air-drying, the filters were baked for 2 h at 80 °C in an oven, and then were pre-hybridized with sheared salmon sperm DNA (0.1 mg·mL^−1^) for 2 h at 65 °C in a buffer containing 40 mM Na_2_HPO_4_, 10 mM NaH_2_PO_4_, 1% bovine serum albumin (BSA), and 7% SDS, before addition of ^32^P labeled [43] probes. After overnight hybridization at 65 °C, the membranes were washed with 2× SSC (300 mM NaCl, 50mM citric acid; pH 7.0) with 0.1% SDS, 1× SSC with 0.1% SDS, and 0.5× SSC with 0.1% SDS, respectively, for 15–20 min. After washing, the membranes were exposed to X-ray film at −80 °C for 2 to 3 days.

### 3.6. Descending Pooling

**Figure 2:**
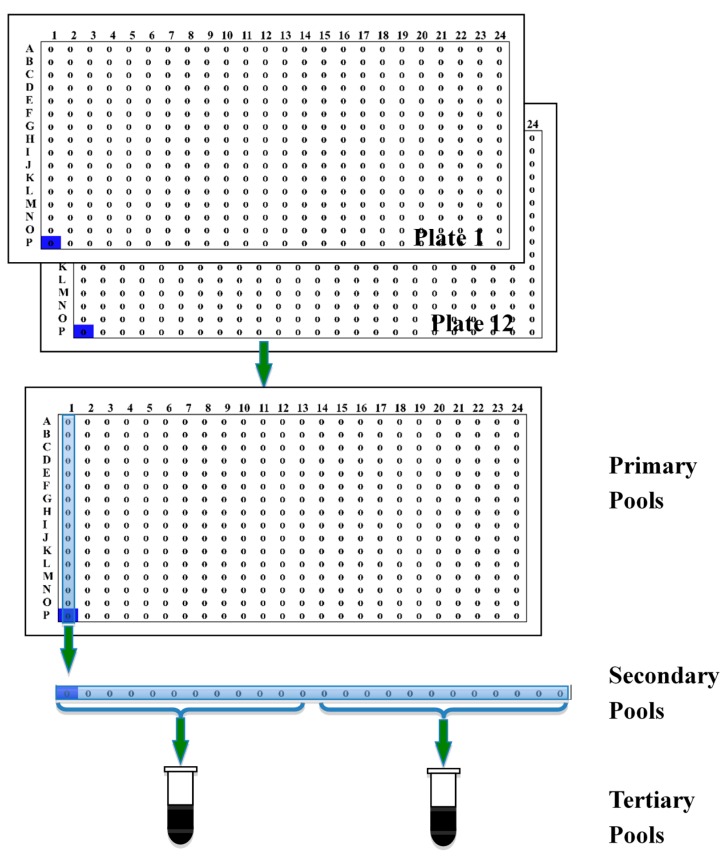
Illustration of descending pooling system. Each of the tertiary pools consists of DNA of 12 secondary pools. Each of the secondary pools was a mixture of the 12 primary pools arrayed in the same column on a 384-well plate. The primary pools were made by combining cells at the corresponding positions on twelve 384-well plates.

To reduce the workload of BAC library screening, we designed a descending-pool system. The simple pooling procedure is shown in Figure 2A tertiary pool was made by combining DNA from cells of 12 secondary pools. The plasmid DNA of the secondary pools was extracted from 3 mL cultures. The DNA was dissolved in 20 μL TE and diluted in 1:100 with TE before the tertiary pools were made. Pooling twelve 384-well plates using this strategy resulted in two tertiary pools, each with 2304 clones; 24 secondary pools each with 192 clones; and 384 primary pools, each with 12 clones.

For screening, PCR was first performed with the tertiary DNA. When a positive signal was obtained for the pool, the DNAs of the individual secondary pools constituting the tertiary pool were amplified. Then the DNAs of the corresponding primary pools and individual clones were extracted for PCR screening.

### 3.7. Library Screening

Markers used in library screening are listed in Table 3. PCR amplification was performed by addition of approximately 2 μL diluted plasmid DNA or 10 ng genomic DNA to a 25 μL cocktail containing 1× PCR buffer, 37.5 nmol Mg^2+^, 5 nmol dNTPs, 1 U Taq DNA polymerase (Promega, Madison, WI, USA), and 5 pmol forward and reverse primers, respectively. PCR was performed using the following procedure: three minutes at 94 °C followed by 36 cycles of 30 s at 94 °C, 30 s at TM for annealing and 30–60 s at 72 °C for extension with a final extension step of 5 min at 72 °C. Genomic DNA was used as positive control. PCR products were separated by electrophoresis on 1%–2% (*w*/*v*) agarose gel or by 8% polyacrylamide gel electrophoresis.

To estimate the genome coverage by PCR, lanes showing target bands comparable to positive controls were assigned as positive pools. The number of positive pools was equal to the number of positive clones of the corresponding marker [22,28].

### 3.8. Comparison between 6D and Descending Pooling Strategy

**Figure 3:**
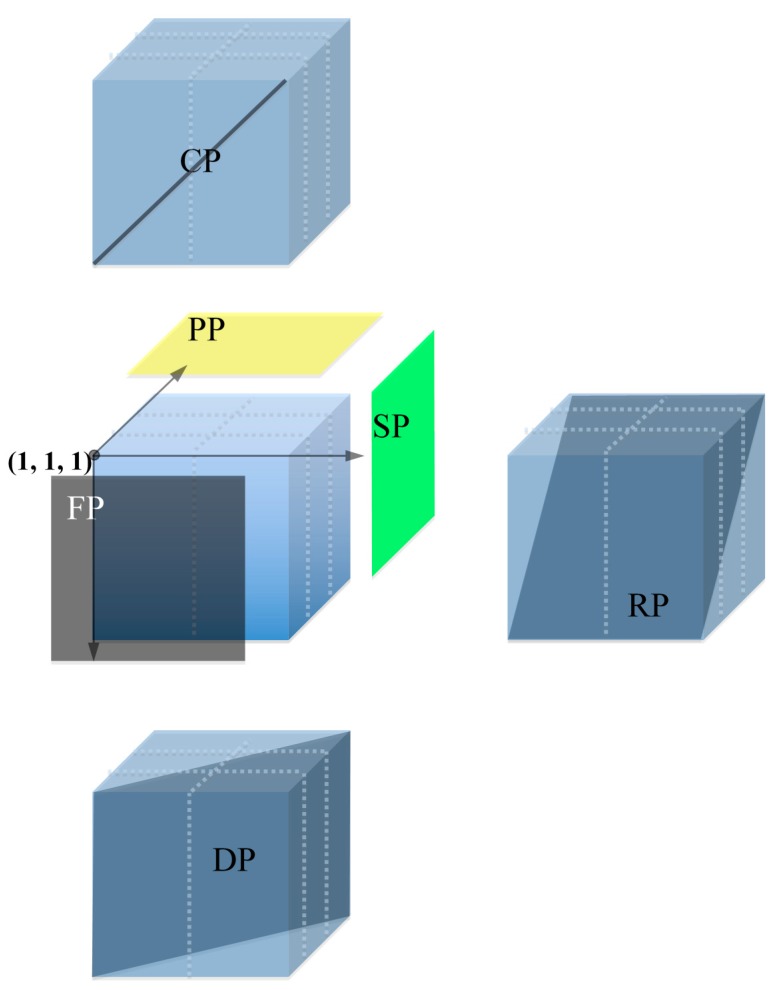
Diagram of 6D pools. Grid made by 288 384-well plates was arranged in 48 layers × 48 rows × 48 columns containing 110,592 clones. Plate pool (PP), face pool (FP), side pool (SP), row pool (RP), column pool (CP), and diagonal pool (DP) were generated as shown, each pool contains 2304 clones.

The Six Dimensional (6D) pools were made as described by Klein *et al.* [23]. A total of 288 384-well plates (chosen from library Wangshuibai-*H*; average insert size 102 kb) were arrayed in a grid containing 48 layers × six plates/layer. The six plates of each layer were arranged in a 2 × 3 matrix (Figure 3). As each 384-well plate was in an array of 24 column × 16 rows, the cube was 48 rows (3 × 6 rows/plate) × 48 columns (2 × 24 columns/plate) × 48 layers. Every clone was given a unique address relative to the axes of the cube, (*x*, *y*, *z*). The *x*-axis was parallel to the row line of the plates, the *y*-axis was vertical to the row line and parallel to the row line of the plates, the *z*-axis was vertical to the *x-* and *y*-axis. Thus, the initial clone located at the near top-left corner was (1, 1, 1) as shown by the black point in Figure 3. The 6D pools include Plate Pool (PP), Face Pool (FP), Side Pool (SP), Row Pool (RP), Column Pool (CP), and Diagonal Pool (DP).

PP, FP, and SP were defined as a set of clones in a plane sharing the same values of *z*-axis, *y*-axis, and *x*-axis, respectively. RP was constructed as follows: BAC clones in row (*y*, *z*) were pooled into RP*_y_*_+ *z* − 1_. To keep the number of clones in each pool constant (2304 clones per pool), wrapping occurred. That is, when *y* + *z* > 49, then 48 was subtracted to get RP*_y _*_+ *z* − 49_ (*i.e.*, BACs in Row 2 Plane 1 and BACs in Row 1 Plane 2 were also together with Row 3 Plane 48 in RP_2_). CP was made as well as RP. BACs in the same column (*x*, *z*) were pooled into CP*_x_*_+ *z* − 1_, and wrapped as the same as RP. Clones in column (*x*) and row (*y*) of all 48 planes were in DP*_x_*_+ *y* − 1_. Forty-eight was subtracted when *x* + *y* > 49 to give the correct pool number. All pools were started from the plane or line that contained the initial point. That is, clone (*x*, *y*, *z*) was in PP*_z_*, FP*_y_*, SP*_x_*, RP*_y_*_+ *z* − 1/(49)_, CP*_x_*_+ *z* − 1/(49)_ and DP*_x _*_+ *y* − 1/(49)_. Each kind of pool has 48 members, thus there were 6D 288 pools.

The same 288 384-well plates were also constructed for descending pools. Plasmids of column pools were isolated and mixed as super pools.

Plasmids of all pools were extracted and diluted as described above and used as templates for PCR amplification. Two SSR markers, WMC413 and BARC95, were used to screen 288 6D pools and 48 descending pools that were constructed from the 288 384-well plates. For descending pools, we amplified the tertiary pools only. The secondary pool was based on the assumption that lanes showing target bands comparable to positive controls were assigned as positive pools.

## 4. Conclusions

The BAC libraries of three disease-resistant germplasms, Wangshuibai, TA2026 and TA2033, represent a new resource for wheat genome research. The three libraries are being employed in genetic map saturation, cloning of disease resistance, and agronomic traits genes [9,46,47,48] as well as in the study of wheat genome organization and evolution. We developed an improved high efficiency descending pooling system to simplify the process of BAC clone screening. The descending pooling system saves more than 68% of PCR reactions and 80% of time spent on pool preparation.
